# Antioxidant and Antivenom Potential of an Essential Oil, 4-(2-Oxo-propyl)-cyclopentane-1,3-dione, and Allantoin Derived from the Polyherbal Combination of *Aristolochia indica* L. and *Piper nigrum* L.

**DOI:** 10.1155/2022/4797884

**Published:** 2022-03-07

**Authors:** Dhivya Sivaraj, Ray J. Butcher, Jerry P. Jasinski, Saikumar Sathyanarayanan, Revathi Ponnusamy, Sreeja Puthanpura Sasidharan, Kasipandi Muniyandi, Parimelazhagan Thangaraj, Karuppusamy Arunachalam

**Affiliations:** ^1^Bioprospecting Laboratory, Department of Botany, Bharathiar University, Coimbatore 641046, Tamil Nadu, India; ^2^Inorganic and Structural Chemistry, Howard University, Washington, DC 20059, USA; ^3^Physical and Structural Chemistry, Department of Chemistry, Keene State College, 229 Main Street, Keene, NH 03435-2001, USA; ^4^Department of Botany, PSG College of Arts and Science (Autonomous), Coimbatore 641014, India; ^5^Department of Botany, Kongunadu Arts and Science College (Autonomous), GN Mills, Coimbatore 641029, India; ^6^Key Laboratory of Economic Plants and Biotechnology and the Yunnan Key Laboratory for Wild Plant Resources, Kunming Institute of Botany, Chinese Academy of Sciences, Kunming 650201, China

## Abstract

The goal of this study was to identify new compounds from a methanol extract of a polyherbal combination of *Aristolochia indica* L. and *Piper nigrum* L. (MECAIPN), two traditional medicinal plants used to cure envenomation, as well as to assess their antioxidant and antivenom properties. MECAIPN yielded EA1 (an essential oil), AA2 (4-(2-oxo-propyl)-cyclopentane-1,3-dione), and W3 ((2,5-dioxo-imidazolidin-4-yl)-urea) (Allantoin). Although EA1 had stronger radical scavenging activity, AA2 had higher DPPH and ferric ion radical scavenging activity, and W3 had higher molybdenum ion radical scavenging activity due to being a single molecule, the binding investigation revealed that EA1 has a greater Stern–Volmer quenching constant (Ksv) than AA2 and W3. Synchronous measurements indicated that EA1, AA2, and W3 bind to tryptophan and tyrosine residues in venom, causing denaturation of the secondary structure of the residue. Finally, the current study concludes that EA1 has more therapeutic antivenom potential, which could be related to the synergism of chemicals found in it. When it came to single compounds, AA2 had stronger antioxidant and antivenom capabilities than W3. To understand the mechanism of action and manufacture the green antivenom medication, more testing of the EA1 and compounds remains required.

## 1. Introduction

The genus “*Scolopendra*” is a venomous terrestrial arthropod whose envenomation has a negative impact on human health due to hypersensitivity. Pain, giddiness, sudden myocardial infarction, and skin-related disorders such as itching, erythema, blemishes, necrosis, aureole, and wound development are all symptoms of hypersensitive reactions. Metalloproteases, hyaluronidase, and phospholipase A2 are among the enzymes in the venom, as are CAP protein, ion channel modulators, cardiotoxin, myotoxin, and cytotoxin, among others. Envenomation also induces oxidative stress by promoting the production of damaging free radicals and damaging macromolecules (lipids, proteins, and nucleic acids). Many illnesses, including as inflammation, cancer, metabolic problems, and aging, are caused by changes in macromolecule structure and function [1]. The medicinal plant is used in the Indian traditional medical systems “Ayurveda and Siddha,” which has the capacity to heal life-threatening disorders. Both systems favor “polyherbal formulation” over uniherbal medication to gain additional therapeutic efficiency through the synergism process [2, 3]. Oraon tribes of Latehar district, Jharkhand, employ the medicinal plants *A. indica* (root) and *P. nigrum* (seed) to cure envenomation [1, 4]. The Malayali tribes of the Yercaud hills ingest *A. indica* root orally for 1 week to cure centipede envenomation [5]. Chittoor district (Andhra Pradesh) uses *P. nigrum* seeds to cure toxic bites [6]. In this regard, a previous study (published in Journal of Biomedicine and Pharmacotherapy) found that a methanol extract of a combination of *A. indica* and *P. nigrum* (MECAIPN) was more effective than individual plant extracts and the standard drug “Medrol” against *Scolopendra morsitans* L. envenomation effects [1]. As a result, the current study aims to isolate new compounds from MECAIPN and assess their antioxidant and antivenom properties.

## 2. Materials and Methods

### 2.1. Chemicals

The solvents used are as follows: methanol, dimethyl sulfoxide (DMSO), chloroform, ethyl acetate, acetic acid, and water, as well as 2,2-diphenyl-1-picrylhydrazyl (DPPH), silica gel, phosphate buffer, ascorbic acid, 2,4,6-tri(2-pyridyl)-s-triazine (TPTZ), ferrous sulphate, and butylated hydroxyltoluene (BHA). Analytical-grade chemicals and solvents were employed throughout.

### 2.2. Extraction

Botanical Survey of India (BSI), Coimbatore, Tamil Nadu, validated the taxonomic identification of *A. indica* (root bark) and *P. nigrum* (seed), which were collected during the month of June (early morning 7.30 am) at Dharapuram (GPS, coordinates: 10° 43′ 58.566″ N and 77° 31′ 18.5268″ E) and Ooty (coordinates: 11.4102° N and 17.6950° E), Tamil Nadu, India (BSI/SRC/5/23/2014-15/Tech.1253 and 1457, respectively). Both powders (CAIPN) were combined in a 1 : 1 ratio (100 g) and extracted for 60 h with Soxhlet at 65°C and 400 mL of methanol. The extract was then concentrated using a rotary vacuum evaporator (Equitron, India) to eliminate the rest of the solvent, and thereafter the extract was air-dried, weighed, and stored in desiccators.

### 2.3. Collection of Venom

*Scolopendra morsitans* was collected in July, Dharapuram district, Tamil Nadu, India, and recognized as *S. morsitans* L. by the Zoological Survey of India (ZSI), Kerala (Reg. no. ZSI/WGRC/IR/INV/4152). The centipedes were kept in containers with air holes, soil, and litter items to keep them alive. Spraying water on the soil kept the moisture content up. The termites were offered as a source of nutrition. Venom was collected by activating the venom glands with an electrical device that had a frequency of 128 Hz, a voltage of 10 to 20 V, and a pulse width of 2 to 4 ms. Each milking occurred 2 weeks after the previous milking. The collected venom was stored at −20°C [7].

### 2.4. Column Chromatography and Characterization

Based on earlier research, MECAIPN was chosen for chemical isolation. MECAIPN (50 g) was adsorbed on activated silica (60–120 mesh) while the methanol was continuously stirred. The column (59 × 4.5 cm) was packed with activated silica gel (60–120 mesh) in petroleum ether (stationary phase) using the wet packing method, and it was eluted with 600 mL of mobile phase containing various solvents such as petroleum ether (100%), petroleum ether (A): chloroform (B) (5–100%), chloroform (A): ethyl acetate (B) (5–100%), ethyl acetate (A): methanol (B) (5–100%) [in order to improve the polarity of the mobile phase, the concentration of “A” was reduced from 95, 90, 85, and 80 to 0%, and the concentration of “B” was raised by 5, 10, 15, 20, 25 up to 100%], acetic acid (1 and 2% in distilled water), and distilled water (100%). The elution volume was 10 mL and the flow rate was 3 mL/min. On a precoated silica gel 60 F254 TLC (Thin layer chromatography) plate, TLC profiling was performed (Merck, Germany). In a saturated chromatographic chamber, the chromatogram was created. The produced plate was examined in an ultraviolet (UV) and iodine chamber. The formula (*R*_*f*_ = distance travelled by the solute/distance travelled by the solvent) was used to get the *R*_*f*_ value of the location. The elution fractions with comparable chemical signatures were grouped together based on TLC findings. From the 4 : 6 (ethyl acetate : methanol) elution, the oil EA1 (4 mL) was recovered. From 1% acetic acid in water and 100% water, the colorless crystals AA2 (4.65 g) (water : methanol : acetic acid in 8 : 1 : 1; *R*_*f*_: 0.85) and W3 (1.32 g) (water : acetic acid in 9.9 : 0.1; *R*_*f*_: 0.11) were produced [8].

### 2.5. Determination of Free Fatty Acid Content and an Acid Value of EA1

One milliliter of EA1 was combined with a neutral solvent (25 mL ether, 25 mL 95% ethanol, and 1% phenolphthalein solution) and titrated against 0.1 N potassium hydroxide for 15 sec until the pink color remained persistent. Using equation (1) mL N/10 KOH (potassium hydroxide) = 0.028 g oleic acid, the free fatty acid was estimated as oleic acid [9The acid value is calculated using the formula, Acid value (mg KOH/mL) = T × N × MW/Volume of EA1 (mL) Where, T = Titre value, N = Normality of KOH, and MW = Molecular weight of KOH.

### 2.6. Determination of Saponification Value of EA1

In a flask, 2 mL of EA1 was weighed. The alcoholic KOH (25 mL) was pipetted into the mixture and allowed to drain for approximately 1 min. A blank determination was made and measured at the same time as the sample. For full saponification, a condenser was attached to the flask, and the combined sample was allowed to boil gently and continuously for 45 min. Thereafter the flask and condenser were cooled, but not enough to form a gel. After disconnecting the condenser, 1 mL of phenolphthalein indicator was added to the flask's contents. With 0.5 N HCL (hydrogen chloride), the solution was titrated until the pink color faded completely [10]. Saponification value was determined using the formula saponification value = (*B* – *S*) × 56.1 × *N*/weight of EA1 (g), where *B* is the blank titre value, *S* is the sample titre value, and *N* is the HCL normality.

### 2.7. Determination of Molecular Weight of EA1

From saponification and an acid value, the molecular weight [11] of EA1 was determined as MW = 168300/SV AV, where MW represents the oil's molecular weight, SV represents the saponification value, and AV represents the acid value.

### 2.8. Gas Chromatography-Mass Spectrum (GC-MS) Analysis of EA1

Shimadzu GC-MS, QP2010S, was used for the GC-MS analysis. Compound separations were carried out on an Rxi-5Sil MS column with a length of 30 m, a diameter of 0.25 mm, and a thickness of 0.25 *µ*m. The chromatographic and mass spectrometric data were processed using GC-MS Solutions software. The temperature in the GC oven was kept at 700°C for 5 min, then steadily increased to 2900°C min^−1^ at a rate of 50°C min^−1^ for another 5 min. The oil sample was divided at a 1 : 16 splitting ratio and injected in the split mode. As a carrier gas, helium was employed at a constant flow rate of 1 mL min^−1^. The electron impact mode of the mass selective detector was used, with an electron energy of 70 eV. The obtained GC-MS profile was examined by comparing the chromatogram to commercially available standards using the NIST (National Institute of Standards and Technology) 11 and WILEY 8 libraries [12].

### 2.9. Fourier Transform Infrared Spectroscopy (FT-IR) Analysis

To make transparent sample discs, EA1 (0.5 mL) and 10 mg of AA2 and W3 crystal powder were encapsulated in 100 mg of KBr pellet. To identify the distinctive peaks and their functional groups, the powdered sample was put into an FT-IR (Fourier transform infrared) spectroscopy (Perkin Elmer Spectrum GX) in the (4000–400) cm^−1^ range. The FT-peak IR's values were recorded. For spectrum confirmation, the procedure was done twice [13].

### 2.10. Nuclear Magnetic Resonance Spectroscopy (NMR)

The signal processing information base was used to determine the ^1^H-NMR and ^13^C-NMR spectra. At room temperature, ^1^H (−2–15 ppm) and ^13^C (0–220 ppm) NMR were recorded on a Bruker DPX 400 spectrometer (ppm, *J* in Hz) running at 400 MHz ^1^H (−2–15 ppm) and ^13^C (0–220 ppm). Chemical changes were measured in parts per million (ppm). The internal standard was tetramethylsilane (TMS). Ten milligrams of compounds were dissolved in a maximum of 2 mL of DMSO, which was chosen as the solvent of choice due to the compounds' solubility. Clean Pasteur pipettes were used to pipette the impurity-free solutions into NMR tubes for examination.

### 2.11. Energy Dispersive X-Ray Analysis (EDX)

The EDX was used to determine the elemental makeup of the AA2 and W3 (Brukers). Gold sputtering was used to cover the compounds prior to analysis. Low electron energies of 1 keV to 10 keV were used to capture secondary electron pictures.

### 2.12. Single-Crystal X-Ray Diffraction Analysis (Sc-XRD)

The X-ray crystallographic investigation was performed on a clear vivid white columnar-like specimen with approximate dimensions of 0.165 mm × 0.201 mm × 0.365 mm (AA2). The X-ray intensity measurements were collected in a Bruker D8 Quest over a 3:04-h room temperature exposure. A narrow-frame method was used to merge the frames using the Bruker Saint Software package. The multi-scan approach was used to adjust for absorption effects in the data (SADABS). The solvent Apex II program was used to solve the structure.  Table 1 shows the data gathering and refining procedure for AA2. For the X-ray crystallographic study, a clear bright white columnar-like specimen with approximate dimensions of 0.558 × 0.168 × 0.030 mm^3^ (W3) was placed on a Mitgencryoloop in random orientation (Bruker AXS SMART APEX CCD).  Table 2 shows the specifics of W3's data collecting and refining procedures.

### 2.13. In Vitro Antioxidant Assay

#### 2.13.1. DPPH^●^ Scavenging Activity

Approximately 3 mL of 0.1 mM methanolic DPPH solution was added to the aliquots of the samples and standards (BHT) and vigorously shaken. The negative control was made by mixing 100 *µ*L methanol with 3 mL of 0.1 mM DPPH methanol solution. At 27°C, the tubes were left to stand for 30 min. The sample's absorbance was measured at 517 nm and represented as an IC_50_, which was the sample concentration necessary to inhibit 50% of DPPH concentration [8].

#### 2.13.2. Ferric Reducing Antioxidant Power Assay

A quantity of 2.5 mL of 10 mM TPTZ in 40 mM HCL, 2.5 mL of 20 mM FeCl_3_.6H_2_O, and 25 mL of 0.3 M acetate buffer were combined to make the FRAP reagent (pH 3.6). The FRAP reagent (900 *µ*L) was combined with 270 *µ*L of distilled water and 100 *µ*L of test material after being newly produced and incubated at 37°C. The test sample was diluted to 1/34 in the reaction mixture at the end. In a water bath, the test samples and reagent blank were incubated for 30 min at 37°C. A spectrophotometer was used to measure the absorbance at 593 nm against the reagent blank at the conclusion of the incubation. As a baseline, BHT was employed. For the calculations, a methanolic solution with known Fe(II) concentrations ranging from 500 to 4000 mM (FeSO_4_·7H_2_O) was used. The equivalent concentration was estimated as the antioxidant concentration that increased the absorbance in the FRAP experiment to the predicted absorbance value of 1 mM Fe(II) solution [8].

#### 2.13.3. Phosphomolybdenum Activity

In a test tube, triplicates of 100 *µ*L of samples were mixed with 3 mL of reagent solution (0.6 M sulphuric acid, 28 mM sodium phosphate, and 4 mM ammonium molybdate) and varied quantities of standard (ascorbic acid in 1 mM dimethyl sulphoxide). The test tubes were incubated for 90 min in a water bath at 95°C. The absorbance of the combination was measured at 695 nm against the reagent blank after the samples had cooled to room temperature. A typical blank solution had 3 mL of reagent solution and water in the place of the sample, and it was incubated at the same temperature as the other samples. As a control, the synthetic antioxidant BHT was utilized. The findings were given as mean values in mg of ascorbic acid equivalents (AAE) per gram of the sample [8].

### 2.14. Venom Protein Binding Studies

The venom was dissolved in phosphate buffer (*µ*L/mL) (pH 7.2). For the venom protein binding investigation, the excitation wavelength of venom was measured at 280 nm and the emission wavelength was measured at 346 nm. For all of the trials, the excitation and emission slit widths and scan speeds were kept constant. For all of the studies, a concentrated stock solution of the samples was generated by dissolving the relevant materials in DMSO, phosphate buffer, and diluting appropriately with deionized water to the needed concentrations (percentage of DMSO (v/v) in the final solution). Titrations were carried out manually with the addition of the samples (10–100 *µ*M) using a micropipette. The same concentration of venom and samples was utilized for synchronous fluorescence spectra, and the spectra were taken at two distinct Δ*λ* values (difference between the excitation and emission wavelengths of venom), such as 15 and 60 nm. A 1 cm quartz cell on a JASCO FP 6600 spectrofluorimeter [14] was used for fluorescence and synchronous measurements.

The quenching ability was evaluated by the Stern–Volmer equation:

(1) $$\begin{array}{c} \frac{I_{o}}{I_{\text{corr}}}=K_{\text{SV}}\left[Q\right]+1, \end{array}$$

where *I*_*o*_ is the emission intensity in the absence of samples, *I*_corr_ is the corrected emission intensity in the presence of the sample, *K*_sv_ is the quenching constant, and [*Q*] is the concentration of the sample.

In order to correct the inner filter effect, the following equation is used [15]:

(2) $$\begin{array}{c} I_{\text{corr}}=I_{\text{obs}}*10^{\left(A_{\text{exc}}+A_{\text{em}}\right)/2}, \end{array}$$

where *I*_corr_ is the corrected fluorescence value, *I*_obs_ is the measured fluorescence value, *A*_exc_ is the absorption value at the excitation wavelength, and *A*_em_ is the absorption value at the emission wavelength. The binding constant (*K*_*b*_) and the number of binding sites (*n*) may be calculated using the static quenching interaction, assuming comparable and independent binding sites are considered in the biomolecule [16]. The Scatchard equation may be used to solve this problem, which is as follows:

(3) $$\begin{array}{c} \log\left[\frac{\left.F_{o}-\;F\right)}{F}\right]=\log\;K_{b\;}+n\;\log\left[Q\right]. \end{array}$$

The number of binding sites per molecule (venom) can be determined by the slope and intercept of a double logarithm regression curve of log [*F*_*o*_ − *F*)/*F*] *versus* log [*Q*], where *K*_*b*_ is the binding constant for the compound–venom protein interaction and “*n*” is the number of binding sites per molecule (venom). At room temperature, the values of “*n*” are roughly equal to 1, indicating that there was just one binding site in the venom for the samples.

### 2.15. Statistical Analysis

Data expressed as mean ± standard deviation were statistically analyzed using SPSS 20.0 by means of one-way ANOVA followed by Duncan's test, significant when *p* < 0.05.

## 3. Results

**Figure 1:**
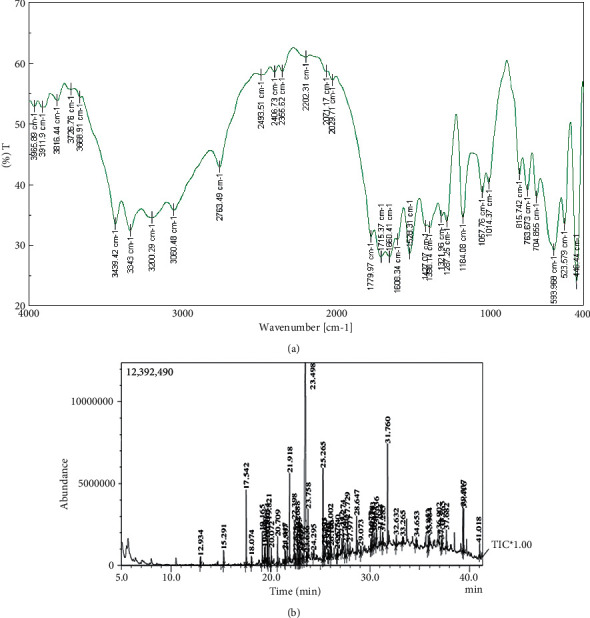
(a) FT-IR spectrum of EA1. (b) GC chromatogram of EA1.

The amount of free fatty acids in the essential oil (EA1) was 43.1 ± 20.18% oleic acid equivalents/g. EA1 has acid and saponification values of 17.28 ± 0.18 mg KOH/mL and 189.3 mg KOH/mL, respectively. EA1 has an average molecular weight of 978.37. FT-IR spectrum showed the presence of functional groups like O-H (3816.44, 3668.91, and 3200.29 cm^−1^), C-H (3060.48, 1608.34, and 1184.08 cm^−1^), and C-O (2355.62, 2202.31, and 1779.97 cm^−1^) (Figure 1(a)). The existence of 63 compounds was discovered using GC-MS (Table 3), and their GC chromatogram is shown in Figure 1(b). Six were the major compounds and they are 1, 5, 5-trimethyl-6-(3-methyl-buta-1,3-dienyl)-cyclohexene (27.74% and RT: 23.498), 1-heneicosanol (4.91% and RT: 31.760), ledol (4.29% and RT: 20.072), E-15-heptadecenol (4.12% and RT: 25.265), heptadecane (3.30% and RT: 17.542), and phenol, 2,4,-bis(1,1, dimethyl ethyl) (3.06% and RT: 19.821)

**Figure 2:**
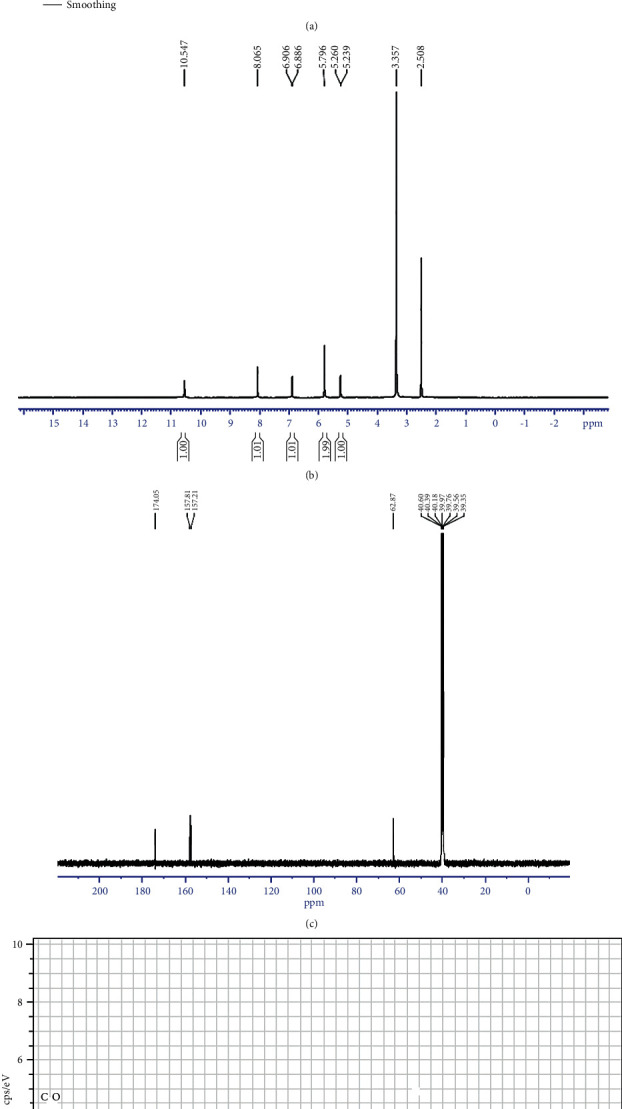
(a) FT-IR spectrum of compound AA2. (b) ^1^H-NMR spectrum of compound AA2. (c) ^13^C-NMR spectrum of compound AA2. (d) EDX spectrum of compound AA2. (e) Chemical structure of compound AA2.

The presence of functional groups such as O-H (3776.62 cm^−1^), H-C=O : C-H (2358.94 and 2856.58 cm^−1^), and C-H (1454.33 and 1371.39 cm^−1^) could be seen in the absorption bands of the FT-IR spectrum (Figure 2(a)). Peaks corresponding to the chemical shift were found at 10.547 ppm (H); 8.065 ppm (C=C-H); 6.906 and 6.886 ppm (H-C=C-); and 5.796, 5.260, and 5.29 ppm (C-OH) on the ^1^H-NMR spectrum (Figure 2(b)). Peaks were found at chemical shifts of 174.05 ppm (CH and CH_2_); 157.41 and 157.21 ppm (C=C); and 62.03 ppm (Figure 2(c)) (CH_2_). In the compound AA2, EDX examination revealed the existence of components such as carbon (C) and oxygen (O) (Figure 2(d)). Tables 4 and 5 exhibit Sc-XRD data for the specified bond lengths and orientations of the compound AA2. This chemical crystallized in the P 1 21/c 1 monoclinic space group.  Table 6 shows the atomic coordinates and corresponding isotropic atomic displacement parameters (Å^2^) of compound AA2.  Table 7 lists the torsion angles (°), whereas Tables 8 and 9 provide the anisotropic atomic displacement parameters (Å^2^) and hydrogen atomic coordinates and isotropic atomic displacement parameters (Å^2^). The molecular structure of the chemical AA2 was determined using these data. The molecule was identified as 4-(2-oxo-propyl)-cyclopentane-1,3-dione using the Chem Draw Ultra program 7.0.1 version and its molecular formula is C_8_H_6_O_3_ (Molecular weight: 150.13 g/mol). Its chemical structure is depicted in Figure 2(e). The compound AA2 was assigned the number 1574042 by the Cambridge Crystallographic Data Centre (CCDC).

**Figure 3:**
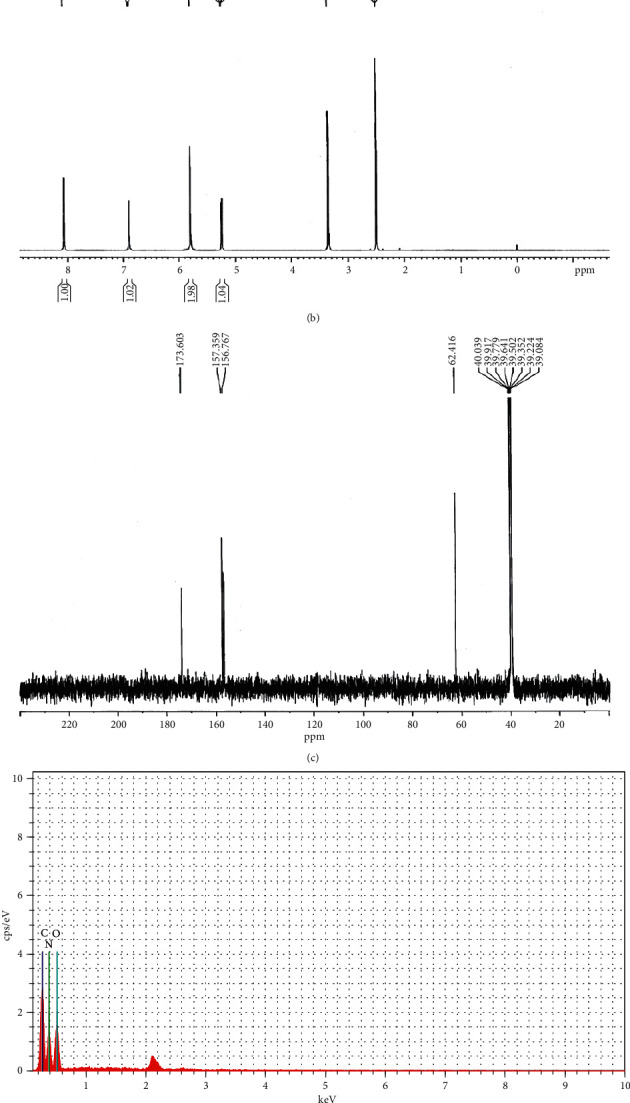
(a) FT-IR spectrum of compound W3. (b) ^1^H-NMR spectrum of compound W3. (c) ^13^C-NMR spectrum of compound W3. (d) EDX spectrum of compound W3. (e) Hydrogen bonding of compound W3. (f) Chemical structure of compound W3.

W3 absorption bands in the FT-IR spectrum are shown in Figure 3(a). O-H (3438.46 and 3345.89 cm^−1^), C=O (1716.34, 1780.94, and 1659.45 cm^−1^), N-H (1659.45 cm^−1^), C-H (1530.24 cm^−1^), and O=C-H (2764.46 cm^−1^) were all found. The ^1^H-NMR and ^13^C-NMR of W3 are shown in Figure 3(b) and 3(c). Peaks at chemical shifts of 8.06, 6.90, 5.79, and 5.25 ppm indicate the presence of (N-H) groups, whereas the peak at 3.35 ppm shows the presence of (C-H) groups. Peaks corresponding to chemical shifts at 173.60, 157.35, 156.76, and 62.41 ppm were found by ^13^C-NMR, indicating the existence of four carbons. The molecule W3 has carbon (C), nitrogen (N), and oxygen (O) in its EDX spectra (Figure 3(d)). Tables 10 and 11 provide Sc-XRD data for the specified bond length and bond angles of compound W3. The chemical W3 formed monoclinic space group P 21/c crystals.  Table 12 shows the distance between hydrogen acceptor and donor, whereas Figure 3(e) shows hydrogen bonding. The atomic coordinates (×10^4^) and comparable isotropic displacement parameters (Å^2^ × 10^3^) described in Tables 13–15 show anisotropic displacement parameters (Å^2^ × 10^3^) and hydrogen coordinates (×10^4^), as well as isotropic displacement parameters (Å^2^ × 10^3^).  Table 16 shows the torsional angles (°) of W3. W3's chemical formula was predicted using these data as C_4_H_6_N_4_O_3_ (molecular weight: 158.13 g/mol).  Figure 3(f) depicts the chemical structure of W3 as predicted by Chem Draw Ultra software 7.0.1 version as (2,5-dioxo-imidazolidin-4-yl)-urea. Compound W3 has a CCDC number of 1574043.

The radical scavenging activity of EA1 was greater than that of AA2 and W3 for DPPH (IC_50_ = 23.17 *µ*g/mL), ferric (23303.57 ± 68.53 Fe(II) E/g of the sample), and molybdenum (709.17 ± 1.51 mg AAE/g of the sample) (Table 17). When compared to EA1, the synthetic antioxidant BHT had stronger radical scavenging activity. However, when it came to single compounds, AA2 was better at scavenging DPPH and ferric ions radicals, whereas W3 was better at scavenging molybdenum radicals.

**Figure 4:**
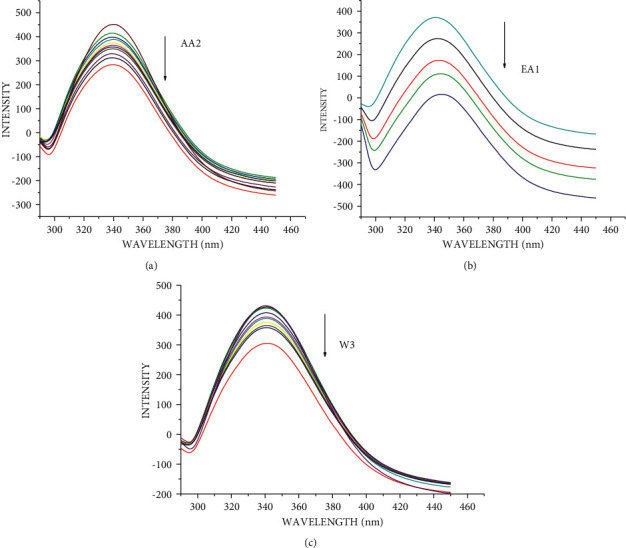
Emission spectra of venom in the presence of increasing concentration of EA1, AA2, and W3. The arrow shows the emission intensity changes upon increasing EA1, AA2, and W3 concentrations. (a) EA1 (an essential oil), (b) AA2 (4-(2-oxo-propyl)-cyclopentane-1,3-dione), and (c) W3((2,5-dioxo-imidazolidin-4-yl)-urea or Allantoin).

**Figure 5:**
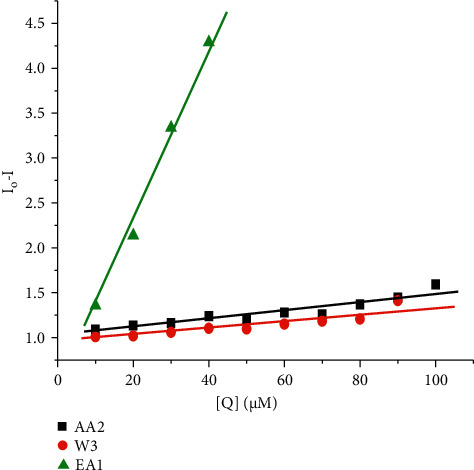
Stern–Volmer plot of fluorescence titration of the EA1, AA2, and W3 with venom. (a) EA1 (an essential oil), (b) AA2 (4-(2-oxo-propyl)-cyclopentane-1,3-dione), and (c) W3((2,5-dioxo-imidazolidin-4-yl)-urea or Allantoin).

**Figure 6:**
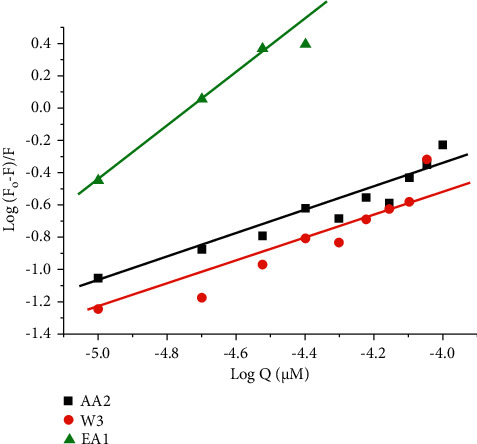
Scatchard plot of fluorescence titration of the EA1, AA2, and W3 with venom. (a) EA1 (an essential oil), (b) AA2 (4-(2-oxo-propyl)-cyclopentane-1,3,-dione), and (c) W3((2, 5-dioxo-imidazolidin-4-yl)-urea or Allantoin).

**Figure 7:**
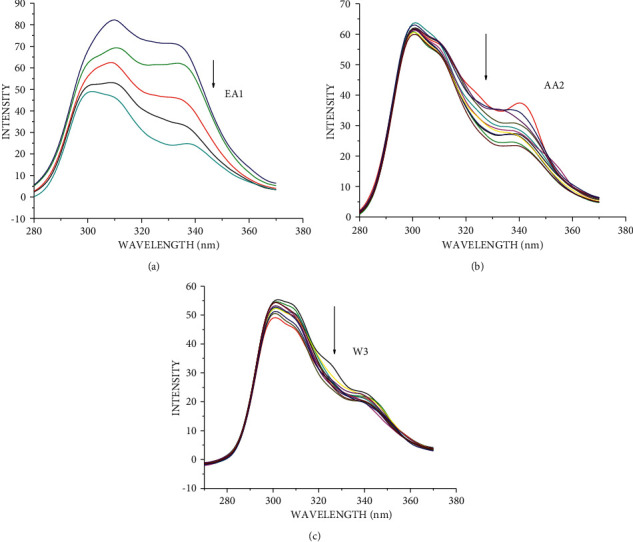
Synchronous spectra of venom in the presence of increasing concentration of EA1, AA2, and W3 for a wavelength difference of Δ*λ* = 15 nm. The arrow shows the emission intensity changes upon increasing concentration of EA1, AA2, and W3. (a) EA1 (essential oil), (b) AA2 (4-(2-oxo-propyl)-cyclopentane-1,3-dione), and (c) W3 ((2,5-dioxo-imidazolidin-4-yl)-urea or Allantoin).

**Figure 8:**
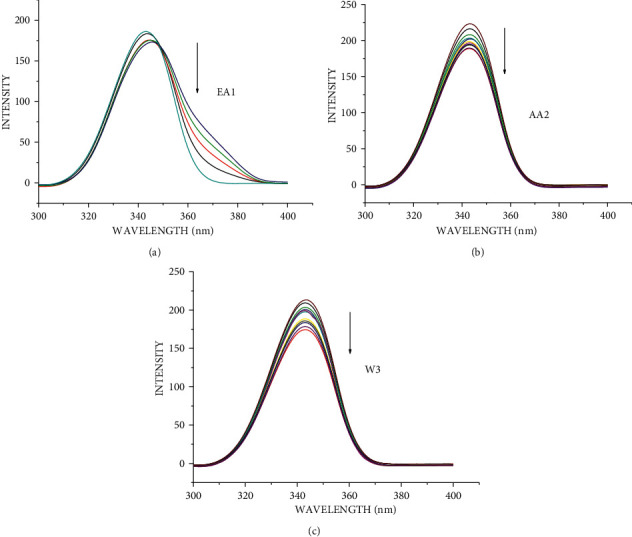
Synchronous spectra of venom in the presence of increasing concentration of EA1, AA2, and W3 for a wavelength difference of Δ*λ* = 60 nm. The arrow shows the emission intensity changes upon increasing concentration of EA1, AA2, and W3. (a) EA1 (essential oil), (b) AA2 (4-(2-oxo-propyl)-cyclopentane-1,3-dione), and (c) W3 ((2,5-dioxo-imidazolidin-4-yl)-urea or Allantoin).

The changes observed on the emission spectra of S. morsitans venom as a result of the addition of increasing concentration of EA1, AA2, and W3 samples (Figures 4(a)–4(c)). The fluorescence intensity of venom at 340 nm, 340 nm, and 341 nm decreased by 37.1%, 29.2%, and 95.5% for the samples AA2, W3, and EA1, respectively, with blueshifts of 1 nm, 1 nm, and redshifts of 4 nm. The quenching constant *K*_sv_ calculated from the plot of *I*_*o*_/*I* against [*Q*] was determined to be 4.78 0.054104 M^−1^, 4.11 0.054104 M^−1^, and 9.99 0.123 105 M^−1^, respectively, according to the Stern–Volmer quenching equation. The capacity of the materials to quench the emission intensity of venom was demonstrated by the linearity obtained in the Stern–Volmer plots of Figure 5 and Table 18. The Scatchard plot (Figure 6) was used to calculate the binding constant (*K*_bin_) and the number of binding sites (*n*), and the findings are provided in Table 18. EA1 had a higher binding constant (9.52 ± 0.08 10^6^) and a larger number of binding site factor (1.479) than both AA2 and W3. There was a drop in the emission intensity of the venom corresponding to tyrosine and tryptophan residues with a red shift after adding the samples (Figures 7(a)–7(c) and 8(a)–8(c)). 4-(2-Oxo-propyl)-cyclopentane-1,3-dione, on the other hand, had higher potential in reducing the intensity of venom emission than (2,5-Dioxo-imidazolidin-4-yl)-urea as a single chemical.

## 4. Discussion

An essential oil, and two colorless crystal compounds such as AA2—4-(2-oxo-propyl)-cyclopentane-1,3-dione—and W3—(2,5-dioxo-imidazolidin-4-yl)-urea (Allantoin)—were extracted using column chromatography in this study. The existence of electronegative elements “oxygen, carbon, and hydrogen,” which is a defining property of an essential oil, was verified by FT-IR analysis of EA1. The volatile oil is also known as an essential oil, and the GC-MS data showed the existence of a volatile antioxidant molecule termed heptadecane, which reduces pro-inflammatory gene expression [17]. Similarly, “ledol,” a sesquiterpene, has anti-inflammatory and NF-*κ*B inhibitory characteristics [18], and phenol, 2,4-bis(1,1, dimethylethyl), is an anti-inflammatory, analgesic, and antioxidant molecule [19]. EA1 also included anti-inflammatory compounds such as globulol [20] and hexadecanoic acid [21]. Analgesic and antipyretic properties of neophytadiene have been observed [22].

The crystal AA2 is a new compound that resembles the structure of 2-methyl-1,3-cyclopentanedione, which was patented (US2016168190) for its composition and to treat traumatic brain injury (TBI); dermatological disorders such as wounds, scars, itching, and ulcers (A61P17/02); joint diseases such as arthritis and arthrosis (A61P19/08); and bone diseases such as rachitis. The crystal W3 is an Allantoin, which is a well-known chemical (PubChem CID: 204) possessing anti-inflammatory, anti-healing, and anti-irritating characteristics. Because of its capacity to heal tiny wounds and promote healthy skin, Allantoin is a common ingredient in anti-acne treatments, sun care products, and clarifying lotions. It is also found in mouthwash, toothpaste, shampoos, lipsticks, other cosmetic lotions, creams, and medicinal items [23].

The capacity of a medication to scavenge free radicals is seen as crucial. Many studies have shown a significant link between antioxidant and antivenom action of substances [24]. EA1 had higher antioxidant activity because it included several antioxidant compounds such as eicosane (C_20_H_42_), neophytadiene (C_20_H_38_), 2-methyltetracosane (C_25_H_52_), heptadecane (C_17_H_36_), nonadecane (C_19_H_40_), tetratricontane (C_44_H_90_), sisterol (C_29_H_50_O), and 1-heptadecanol. These chemicals may act together to stabilize free radicals. In the case of single chemical therapeutic activity, the mechanism of the synergism was not investigated. As a result, the antioxidant potential of AA2 was shown to be much higher than that of W3. As a consequence, the findings imply that EA1 and AA2 may have therapeutic potential in combating the oxidative stress and pathology caused by *S. morsitans* envenomation.

In addition, fluorescence detection is a key method for determining the pharmacological action of (antivenom) substances [25]. The potential of the samples to quench the emission intensity of *S. morsitans* venom was shown by the linearity of the emission spectra [26]. The observed quenching might be ascribed to changes in venom protein secondary structure or denaturation, suggesting sample binding to *S. morsitans* venom protein [27, 28]. The type and amount of the drug–venom protein interaction have a significant impact on the drug's pharmacological activity. The binding characteristics are helpful in determining a drug's pharmacological response and designing a dosing range [29, 30].

*Scolopendra* venom contains the aromatic amino acids tyrosine and tryptophan [31]. Tyrosine is found in venom at 15 nm and tryptophan at 60 nm, according to the research. The blue shift occurs when the AA2 and W3 are added, indicating an increase in hydrophobicity surrounding the fluorophore moiety. With a red shift in synchronous spectra, the EA1 revealed a reduction in emission intensity corresponding to tyrosine and tryptophan residues. In *S. morsitans* venom, red shift indicates an increase in hydrophilicity near the fluorophore [32–34]. The molecular environment in the region of the fluorophore moieties of *S. morsitans* venom was studied using synchronized fluorescence [35]. When comparing the quenching and binding constants, the antivenom activity of EA1, AA2, and W3 was shown to be much higher than that of *S. morsitans* venom. Allantoin, an alkaloid derived from the *A. radix*, was shown to be effective against the haemorrhagic components found in the venoms of *Naja naja atra* and *Bungarus multicinctus* [36]. Allantoin was included to the national library of medicine and toxicology data network because it can be utilized as an antivenom [37]. Due to their greater quenching and binding constants, 4-(2-oxo-propyl)-cyclopentane-1,3-dione (AA2) and an essential oil (EA1) may be more potent antivenom agents than Allantoin (W3).

## 5. Conclusion

Overall, the findings of this investigation backed with the conventional claim linked with *A. indica* and *P. nigrum* literature. It also aids in the promotion of medication usage in the present competitive pharmaceutical industry, which is rife with ineffective medicines. Further research into the interactions of isolated chemicals 4-(2-oxo-propyl)-cyclopentane-1,3-dione, Allantoin, and EA1 with *S. morsitans* venom will aid in a better understanding of the venom neutralization process and the development of a green antivenom medication for human benefit.

## Figures and Tables

**Table 1:** Data collection and refinement program of compound AA2.

Theta range for data collection	2.76 to 27.12°	
Index ranges	−10 ≤ *h* ≤ 10, −6 ≤ *k* ≤ 6, −17 ≤ l ≤ 19	
Reflections collected	8399	
Wavelength	0.71073 Å	
Independent reflections	1355 [*R* (int) = 0.0660]	
Coverage of independent reflections	99.9%	
Absorption correction	Multi-scan	
Max. and min. transmission	0.9790 and 0.9550	
Refinement method	Full-matrix least-squares on *F*^2^	
Refinement program	SHELXL-2014/7 (Sheldrick, 2014)	
Function minimized	Σ *w* (*F*_*o*_^2^ − *F*_*c*_^2^)^2^	
Data/restraints/parameters	1355/0/101	
Goodness-of-fit on *F*^2^	1.080	
Final *R* indices	1134 data; *I* > 2*σ* (*I*)	*R*1 = 0.0810, *wR*2 = 0.2477
	all data	*R*1 = 0.0923, *wR*2 = 0.2612
Weighting scheme	*w* = 1/[*σ*^2^(*F*_*o*_^2^) + (0.1402*P*)^2^ + 1.5335*P*], where *P* = (*F*_*o*_^2^ + 2*F*_*c*_^2^)/3	
Extinction coefficient	0.0800 (200)	
Largest diff. peak and hole	0.638 and −0.380 eÅ^−3^	
R.M.S. deviation from mean	0.113 eÅ^−3^	

**Table 2:** Data collection and refinement program of compound W3.

Theta range for data collection	5.518 to 71.202°.	
Index ranges	−9 ≤ *h* ≤ 9, −6 ≤ *k* ≤ 5, −13 ≤ l ≤ 17	
Reflections collected	1943	
Independent reflections	1153 [*R* (int) = 0.0181]	
Completeness to theta = 67.684°	99.5%	
Wavelength	1.54184 Å	
Absorption correction	Analytical	
Max. and min. transmission	0.922 and 0.495	
Refinement method	Full-matrix least-squares on *F*^2^	
Refinement program	SHELXL-2014/7 (sheldrick, 2014)	
Data/restraints/parameters	1355/0/101	
Goodness-of-fit on *F*^2^	1.046	
Final *R* indices	[*I* > 2sigma (*I*)]	*R*1 = 0.0403, *wR*2 = 0.1072
	all data	*R*1 = 0.0478, *wR*2 = 0.1148
Weighting scheme	*w* = 1/[*σ*^2^(*F*_*o*_^2^) + (0.1402*P*)^2^ + 1.5335*P*] where *P* = (*F*_*o*_^2^ + 2*F*_*c*_^2^)/3	
Extinction coefficient	0.0800 (200)	
Largest diff. peak and hole	0.229 and −0.208 eÅ^−3^	

**Table 3:** Phytocompounds identified through GC-MS analysis of EA1.

Peak	Retention time	Area (%)	Height (%)	Phytocompounds	Compounds (%)	Base (*m*/*z*)
1	12.934	0.37	0.49	Dodecane	0.37	57.05
2	15.291	0.62	0.91	Pentadecane	0.62	57.05
3	17.542	3.30	4.75	Heptadecane	3.30	57.05
4	18.074	0.34	0.52	Caryophyllene	0.34	93.10
5	19.165	1.59	2.04	*α*-Guaiene	1.59	105.10
6	19.402	0.53	0.84	Nonadecane	0.53	57.05
7	19.449	0.63	1.09	4,5-di-epi-Aristolochene	0.63	105.05
8	19.654	1.34	1.95	Hexadecane	1.34	57.05
9	19.707	0.97	1.48	5,*β*,10,*α*-Eudesma-4(14),11-diene	0.97	105.05
10	19.821	3.06	2.80	Phenol, 2,4-bis(1,1-dimethylethyl)	3.06	191.15
11	20.072	0.89	1.02	Cubedol	0.89	161.15
12	20.709	1.30	1.75	Elemol	1.30	59.05
13	21.447	0.55	0.79	Caryophyllene oxide	0.55	79.05
14	21.507	0.48	0.75	Centene	0.48	55.05
15	21.918	4.29	5.52	Ledol	4.29	69.05
16	22.398	1.87	2.46	Ledene oxide-(I)	1.87	125.10
17	22.525	0.41	0.52	trans-Z-*α*-Bisabolene epoxide	0.41	136.10
18	22.688	2.50	2.13	*δ*-Cadinol	2.50	161.15
19	22.742	1.02	1.18	3-Methyldiadamantane	1.02	187.15
20	22.883	1.34	1.10	*β*-Eudesmol	1.34	59.05
21	22.958	0.56	0.67	2-Butyloxycarbonyloxy-1,1,10-trimethyl-6,9-epidioxydecalin	0.56	57.05
22	23.033	0.98	0.60	Octadecane, 1-chloro-	0.98	57.05
23	23.192	0.79	0.46	Limonen-6-ol, pivalate	0.79	57.05
24	23.498	27.74	12.50	1,5,5-Trimethyl-6-(3-methyl-buta-1,3-dienyl)-cyclohexene	27.74	147.10
25	23.636	0.29	0.42	Heptadecane, 2,6,10,15-tetramethyl-	0.29	57.05
26	23.758	2.39	3.12	Globulol	2.39	81.05
27	24.295	0.26	0.41	7-Acetyl-2-hydroxy-2-methyl-isopropylbicyclo[4.3.0]nonane	0.26	153.10
28	25.265	4.12	5.78	E-15-Heptadecenal	4.12	57.05
29	25.375	0.57	0.92	Octadecane	0.57	57.05
30	25.450	0.86	0.74	3-Hexadecanol	0.86	57.05
31	25.760	0.25	0.40	Ethyl iso-allocholate	0.25	60.00
32	25.822	0.22	0.36	Isoaromadendrene epoxide	0.22	93.10
33	26.002	0.97	1.69	Neophytadiene	0.97	57.05
34	26.106	0.67	0.67	Dihydrophytol	0.67	57.05
35	26.675	0.38	0.46	Tricosane	0.38	57.05
36	26.740	0.73	0.99	2-Methyltetracosane	0.73	57.05
37	27.142	0.66	0.87	Cholest-22-ene-21-ol,3,5-dehydro-6-methoxy-, pivalate	0.66	57.05
38	27.274	0.90	1.46	Eicosane	0.90	57.05
39	27.490	0.68	0.85	Hexadecanoic acid, methyl ester	0.68	74.05
40	27.729	2.22	2.21	1,1-Dimethyldecahydronaphthalene	2.22	151.10
41	27.977	0.41	0.73	2-Bromotetradecane	0.41	149.00
42	28.647	2.05	2.40	1-Octadecene	2.05	57.05
43	29.073	0.37	0.51	Tridecanal	0.37	57.05
44	30.077	0.50	0.69	1-Decanol, 2-octyl-	0.50	57.05
45	30.178	0.83	1.03	Methyl 10-trans,12-cis-octadecadienoate	0.83	67.05
46	30.293	1.56	1.21	9-Octadecenoic acid (z)-, methyl ester	1.56	55.05
47	30.457	0.72	0.87	Phytol	0.72	71.05
48	30.636	0.87	1.26	Docosane	0.87	57.05
49	31.044	0.80	0.84	Ethyl 9,12-hexadecadienoate	0.80	81.05
50	31.285	0.92	0.91	2-Methylhexacosane	0.92	57.05
51	31.760	4.91	6.44	1-Heneicosanol	4.91	57.05

**Table 4:** Bond length of compound AA2.

Elements	Bond length
O1-C2	1.250 (4)
O3-C6	1.222 (5)
C1-C2	1.338 (5)
C1-H1B	0.93
C5-C6	1.336 (5)
C5-H5	0.93
C7-C6	1.395 (5)
C3-C2	1.353 (5)
O2-C8	1.208 (4)
C7-C8	1.361 (5)
C1-H1A	0.93
C8-C4	1.536 (5)
C3-H3	0.93
C5-C4	1.462 (5)
C7-H7	0.93
C3-C4	1.422 (4)
C4-H4	0.98

**Table 5:** Bond angle of compound AA2.

Elements	Bond angle
C8-C7-C6	111.6 (3)
C6-C7-H7	124.2
C2-C3-H3	119.6
C6-C5-C4	112.3 (3)
C4-C5-H5	123.8
C2-C1-H1B	120.0
C2-C1-H1A	120.0
H1A-C1-H1B	120.0
C3-C4-C5	115.9 (3)
O1-C2-C1	122.6 (3)
C1-C2-C3	117.3 (3)
O2-C8-C4	126.7 (3)
O3-C6-C5	127.8 (4)
C5-C6-C7	108.1 (3)
C3-C4-C8	113.7 (3)
O1-C2-C3	120.0 (3)
O2-C8-C7	126.6 (3)
C5-C4-C8	100.9 (3)
C3-C4-H4	108.6
C8-C4-H4	108.6
C8-C7-H7	124.2
C2-C3-C4	120.8 (3)
C4-C3-H3	119.6
C6-C5-H5	123.8
C7-C8-C4	106.6 (3)
O3-C6-C7	124.1 (3)
C5-C4-H4	108.6

**Table 6:** Atomic coordinates and equivalent isotropic atomic displacement parameters (Å^2^) of compound AA2.

	*x*/*a*	*y*/*b*	*z*/*c*	*U* (eq)
O1	0.7643 (3)	0.8942 (5)	0.75373 (18)	0.0358 (8)
O2	0.9293 (3)	0.6791 (6)	0.57912 (18)	0.0370 (8)
O3	0.3646 (3)	0.7877 (6)	0.5629 (2)	0.0429 (8)
C7	0.6512 (4)	0.7739 (6)	0.5521 (2)	0.0234 (8)
C3	0.7878 (4)	0.4585 (6)	0.7473 (2)	0.0226 (7)
C5	0.5341 (4)	0.5166 (7)	0.6502 (2)	0.0271 (8)
C1	0.8927 (4)	0.6810 (7)	0.8725 (2)	0.0280 (8)
C2	0.8141 (4)	0.6876 (7)	0.7904 (2)	0.0286 (8)
C8	0.7836 (4)	0.6476 (7)	0.5933 (2)	0.0289 (8)
C6	0.5008 (5)	0.6990 (7)	0.5876 (2)	0.0318 (9)
C4	0.7115 (4)	0.4498 (7)	0.6583 (2)	0.0302 (8)

*U* (eq) is defined as one-third of the trace of the orthogonalized *Uij* tensor.

**Table 7:** Torsion angles (°) of compound AA2.

Elements	Torsion angles (°)
C4-C3-C2-O1	3.3 (5)
C6-C7-C8-O2	−177.2 (3)
C4-C5-C6-O3	176.9 (4)
C8-C7-C6-O3	178.9 (4)
C2-C3-C4-C5	−70.0 (5)
C6-C5-C4-C3	128.1 (3)
O2-C8-C4-C3	51.7 (5)
O2-C8-C4-C5	176.5 (4)
C4-C3-C2-C1	−177.4 (3)
C6-C7-C8-C4	5.0 (4)
C4-C5-C6-C7	−2.1 (4)
C8-C7-C6-C5	−2.0 (4)
C2-C3-C4-C8	46.3 (5)
C6-C5-C4-C8	4.8 (4)
C7-C8-C4-C3	−130.6 (3)
C7-C8-C4-C5	−5.8 (4)

**Table 8:** Anisotropic atomic displacement parameters (Å^2^) of compound AA2.

	*U*_11_	*U*_22_	*U*_33_	*U*_23_	*U*_13_
O1	0.0426 (15)	0.0273 (14)	0.0365 (14)	0.0000 (11)	−0.0060 (11)
O2	0.0293 (14)	0.0446 (17)	0.0366 (15)	0.0052 (11)	−0.0018 (11)
O3	0.0308 (15)	0.0506 (18)	0.0465 (16)	0.0092 (13)	−0.0055 (12)
C7	0.0228 (15)	0.0276 (16)	0.0194 (14)	0.0065 (12)	−0.0027 (11)
C3	0.0301 (16)	0.0188 (15)	0.0181 (14)	0.0023 (11)	−0.0069 (11)
C5	0.0199 (15)	0.0334 (18)	0.0276 (16)	0.0089 (13)	−0.0040 (12)
C1	0.0336 (17)	0.0281 (17)	0.0213 (15)	0.0010 (12)	−0.0085 (12)
C2	0.0280 (16)	0.0297 (18)	0.0278 (17)	0.0006 (13)	−0.0010 (13)
C8	0.0297 (17)	0.0309 (17)	0.0256 (16)	-0.0019 (13)	−0.0034 (13)
C6	0.0300 (18)	0.0348 (19)	0.0299 (17)	-0.0011 (14)	−0.0050 (13)

The anisotropic atomic displacement factor exponent takes the form −2*π*2[*h*2*a∗*2*U*11+⋯+2*hka∗b∗U*12].

**Table 9:** Hydrogen atomic coordinates and isotropic atomic displacement parameters (Å^2^) of compound AA2.

	*x*/*a*	*y*/*b*	*z*/*c*	*U* (eq)
H7	0.6598	0.8952	0.5060	0.028
H3	0.8200	0.3047	0.7762	0.027
H5	0.4536	0.4407	0.6847	0.033
H1A	0.9122	0.8342	0.9046	0.034
H1B	0.9278	0.5233	0.8974	0.034
H4	0.7260	0.2752	0.6337	0.036

**Table 10:** Bond length of compound W3.

Elements	Bond length
O(1)-C(1)	1.218 (2)
O(2)-C(2)	1.225 (2)
O(3)-C(4)	1.245 (2)
N(1)-C(1)	1.352 (2)
N(1)-C(2)	1.393 (2)
N(1)-H(1N)	0.87 (2)
N(2)-C(2)	1.336 (2)
N(2)-C(3)	1.460 (2)
N(2)-H(2N)	0.82 (2)
N(3)-C(4)	1.357 (2)
N(3)-C(3)	1.425 (2)
N(3)-H(3B)	0.8600
N(4)-C(4)	1.333 (2)
N(4)-H(4A)	0.8600
N(4)-H(4B)	0.8600
C(1)-C(3)	1.537 (2)
C(3)-H(3A)	0.9800

**Table 11:** Bond angle of compound W3.

Elements	Bond angle
C(1)-N(1)-C(2)	112.04 (15)
C(1)-N(1)-H(1N)	123.8 (14)
C(2)-N(1)-H(1N)	123.8 (14)
C(2)-N(2)-C(3)	112.39 (14)
C(2)-N(2)-H(2N)	124.0 (17)
C(3)-N(2)-H(2N)	123.6 (17)
C(4)-N(3)-C(3)	120.46 (14)
(4)-N(3)-H(3B)	119.8
C(3)-N(3)-H(3B)	119.8
C(4)-N(4)-H(4A)	120.0
C(4)-N(4)-H(4B)	120.0
H(4A)-N(4)-(4B)	120.0
O(1)-C(1)-N(1)	127.20 (16)
O(1)-C(1)-C(3)	126.15 (15)
N(1)-C(1)-C(3)	106.60 (14)
O(2)-C(2)-N(2)	127.76 (17)
O(2)-C(2)-N(1)	124.40 (16)
N(2)-C(2)-N(1)	107.84 (15)
N(3)-C(3)-N(2)	116.20 (14)
N(3)-C(3)-C(1)	113.80 (14)
N(2)-C(3)-C(1)	100.80 (13)
N(3)-C(3)-H(3A)	108.5
N(2)-C(3)-H(3A)	108.5
C(1)-C(3)-H(3A)	108.5
O(3)-C(4)-N(4)	122.81 (16)
O(3)-C(4)-N(3)	120.16 (16)
N(4)-C(4)-N(3)	117.02 (15)

**Table 12:** Distance between hydrogen acceptor and donor of compound W3.

*D*-H…A	*d* (D-H)	*d* (H…A)	*d* (D…A)	<(DHA)
N(1)-(1N)…O(2)#	10.87 (2)	1.98 (2)	2.829 (2)	167 (2)
N(2)-(2N)…O(3)#	20.82 (2)	2.13 (2)	2.923 (2)	160 (2)
N(3)-(3B)…O(3)#	30.86	2.23	2.9115 (19)	136.2
N(4)-(4A)…O(1)#	40.86	2.25	3.006 (2)	145.9
N(4)-(4B)…O(1)#	60.86	2.20	3.023 (2)	161.2
C(3)-(3A)…O(3)#	30.98	2.66	3.212 (2)	116.0

Symmetry transformations used to generate equivalent atoms: #1 −*x* + 1, −*y*, −*z* + 1 #2 −*x* + 1, *y* + 1/2, −*z* + 1/2 #3 *x*, *y* + 1, *z* #4 −*x*, *y* − 1/2, −*z* + 1/2 #5 *x*, −*y* + 1/2, *z* − 1/2 #6 −*x*, *y* + 1/2, −*z* + 1/2.

**Table 13:** Atomic coordinates (×10^4^) and equivalent isotropic displacement parameters (Å^2^ × 10^3^) of compound W3.

	*x*	*Y*	*z*	*U* (eq)
O(1)	703 (2)	3211 (2)	4209 (1)	31 (1)
O(2)	6355 (2)	2121 (3)	4371 (1)	37 (1)
O(3)	2354 (2)	1060 (2)	2461 (1)	30 (1)
N(1)	3488 (2)	2268 (3)	4477 (1)	27 (1)
N(2)	4656 (2)	4841 (3)	3501 (1)	31 (1)
N(3)	2121 (2)	5421 (3)	2526 (1)	26 (1)
N(4)	1081 (2)	3192 (3)	1277 (1)	32 (1)
C(1)	2170 (2)	3514 (3)	4069 (1)	24 (1)
C(2)	4990 (2)	3014 (3)	4126 (1)	27 (1)
C(3)	2886 (2)	5500 (3)	3418 (1)	25 (1)

*U* (eq) is defined as one-third of the trace of the orthogonalized *U*^*ij*^ tensor.

**Table 14:** Anisotropic displacement parameters (Å^2^ × 10^3^) of compound W3.

	*U* ^11^	*U* ^22^	*U* ^33^	*U* ^23^	*U* ^13^	*U* ^12^
O(1)	26 (1)	32 (1)	35 (1)	6 (1)	4 (1)	2 (1)
O(2)	28 (1)	38 (1)	43 (1)	9 (1)	−1 (1)	5 (1)
O(3)	38 (1)	17 (1)	34 (1)	1 (1)	1 (1)	5 (1)
N(1)	30 (1)	25 (1)	26 (1)	5 (1)	2 (1)	3 (1)
N(2)	26 (1)	31 (1)	37 (1)	10 (1)	2 (1)	−4 (1)
N(3)	37 (1)	16 (1)	25 (1)	2 (1)	−2 (1)	3 (1)
N(4)	42 (1)	24 (1)	28 (1)	0 (1)	−5 (1)	−3 (1)
C(1)	31 (1)	20 (1)	22 (1)	−2 (1)	0 (1)	1 (1)
C(2)	30 (1)	23 (1)	28 (1)	−2 (1)	1 (1)	0 (1)
C(3)	28 (1)	17 (1)	29 (1)	−1 (1)	0 (1)	−1 (1)

The anisotropic displacement factor exponent takes the form: −2*π*2[*h*2*a∗*2*U*11+⋯+2*hka∗b∗U*12].

**Table 15:** Hydrogen coordinates (×10^4^) and isotropic displacement parameters (Å^2^ × 10^3^) of compound W3.

	*x*	*y*	*Z*	*U* (eq)
H(1N)	3400 (30)	1020 (50)	4866 (15)	31 (5)
H(2N)	5360 (30)	5500 (50)	3189 (16)	43 (6)
H(3B)	1825	6844	2259	31
H(4A)	908	1779	977	38
H(4B)	754	4653	1049	38
H(3A)	2739	7244	3666	30

**Table 16:** Torsion angles (°) of compound W3.

Elements	Torsion angles (°)
C(2)-N(1)-C(1)-O(1)	177.48 (16)
C(2)-N(1)-C(1)-C(3)	−5.09 (19)
C(3)-N(2)-C(2)-O(2)	−177.39 (17)
C(3)-N(2)-C(2)-N(1)	2.0 (2)
C(1)-N(1)-C(2)-O(2)	−178.42 (17)
C(1)-N(1)-C(2)-N(2)	2.2 (2)
C(4)-N(3)-C(3)-N(2)	70.4 (2)
C(4)-N(3)-C(3)-C(1)	−46.0 (2)
C(2)-N(2)-C(3)-N(3)	−128.17 (16)
C(2)-N(2)-C(3)-C(1)	−4.68 (18)
O(1)-C(1)-C(3)-N(3)	−51.7 (2)
N(1)-C(1)-C(3)-N(3)	130.82 (15)
O(1)-C(1)-C(3)-N(2)	−176.84 (16)
N(1)-C(1)-C(3)-N(2)	5.70 (17)
C(3)-N(3)-C(4)-O(3)	−3.6 (2)
C(3)-N(3)-C(4)-N(4)	177.74 (15)

**Table 17:** Antioxidant activity of EA1, AA2, and W3.

Samples	DPPH radical scavenging activity (IC_50_ *µ*g/mL)	Phosphomolybdenum (mg·AAE/g of the sample)	FRAP (Fe(II) E/g of the sample)
EA1	23.17	709.17 ± 1.51^a^	23303.57 ± 68.53^b^
AA2	159.23	72.87 ± 0.99^d^	7748.91 ± 44.86^c^
W3	182.89	121.71 ± 1.14^c^	351.03 ± 68.53^d^
BHT	3.48	419.73 ± 0.57^b^	45169.83 ± 118.23^a^

EA1: essential oil; AA2: 4-(2-oxo-propyl)-cyclopentane-1,3-dione; W3: (2,5-dioxo-imidazolidin-4-yl)-urea or Allantoin; AAE: ascorbic acid equivalents; Fe(II) E: ferrous ion equivalents; values are mean of triplicate determination (*n* = 3) ± standard deviation. Statistically significant at *p* < 0.05, where a > b > c > d in each row.

**Table 18:** Quenching constant (*K*_sv_), binding constant (*K*_bin_), and the number of binding sites (*n*) for the interaction of EA1, AA2, and W3 with venom.

Compounds	Quenching constant (*K*_SV_/M^−1^)	Binding constant (*K*_bin_/M^−1^)	Number of binding sites (*n*)
EA1	9.99 ± 0.123 × 10^5^	9.52 ± 0.08 × 10^6^	1.479
AA2	4.78 ± 0.054 × 10^4^	4.12 ± 0.07 × 10^2^	0.744
W3	4.11 ± 0.054 × 10^4^	1.27 ± 0.09 × 10^3^	0.892

(*a*) EA1 (an essential oil), (*b*) AA2 (4-(2-oxo-propyl)-cyclopentane-1,3-dione), and (*c*) W3 ((2,5-dioxo-imidazolidin-4-yl)-urea or Allantoin).

## Data Availability

The data used to support the findings of this study are included in the article.

## Abbreviations

AV: Acid value
AA2: Compounds isolated 1% acetic acid in water
AAE: Ascorbic acid equivalents
BSI: Botanical survey of India
BHT: Butylated hydroxytoluene
CAIPN: Combination of *Aristolochia indica* L. and *Piper nigrum* L.
CCDC: Cambridge Crystallographic Data Centre
DPPH: 2,2-Diphenyl-1-picrylhydrazyl
EA1: Oil eluted from 40% ethyl acetate in methanol
FRAP: Ferric reducing antioxidant power
MECAIPN: Methanol extract of the polyherbal combination of *Aristolochia indica* L. and *Piper nigrum* L.
SV: Saponification value
TBI: Traumatic brain injury
TPTZ: 2,4,6-Tri(2-pyridyl)-s-triazine
W3: Compounds isolated from 100% water
ZSI: Zoological survey of India.
